# Cartilage Oligomeric Matrix Protein (COMP): A Biomarker of Arthritis

**DOI:** 10.4137/bmi.s645

**Published:** 2009-02-17

**Authors:** Susan Tseng, A. Hari Reddi, Paul E. Di Cesare

**Affiliations:** Department of Orthopaedic Surgery, UC Davis Medical Center, Sacramento, California, 95817, U.S.A

**Keywords:** cartilage oligomeric matrix protein, arthritis, biomarker

## Abstract

Arthritis is a chronic disease with a significant impact on the population. It damages the cartilage, synovium, and bone of the joints causing pain, impairment, and disability in patients. Current methods for diagnosis of and monitoring the disease are only able to detect clinical manifestations of arthritis late in the process. However, with the recent onset of successful treatments for rheumatoid arthritis and osteoarthritis, it becomes important to identify prognostic factors that can predict the evolution of arthritis. This is especially critical in the early phases of disease so that these treatments can be started as soon as possible to slow down progression of the disease. A valuable approach to monitor arthritis would be by measuring biological markers of cartilage degradation and repair to reflect variations in joint remodeling. One such potential biological marker of arthritis is cartilage oligomeric matrix protein (COMP). In various studies, COMP has shown promise as a diagnostic and prognostic indicator and as a marker of the disease severity and the effect of treatment. This review highlights the progress in the utilization of COMP as a biomarker of arthritis.

## Introduction

Arthritis is a longstanding, debilitating disease that results in serious repercussions on the population. It causes pain, impairment, and disability in patients with increasing injury to the cartilage, synovium, and bone of the joints. Existing methods to diagnose and to monitor the disease are based on late clinical manifestations of arthritis. However, with recent developments of successful treatments for rheumatoid arthritis and osteoarthritis, it becomes important to identify prognostic factors that can predict the evolution of arthritis. This would be of most value in the early phases of the disease so that treatments could be started expeditiously to help slow down progression of the disease. A possible approach to monitor arthritis would be to measure biological markers of cartilage repair and degradation to reflect variations in joint remodeling. One such potential biological marker of arthritis is cartilage oligomeric matrix protein (COMP). This review highlights the progress in the utilization of COMP as a biomarker of arthritis. The disease of arthritis and the need for a biomarker will be discussed, along with COMP and its value as a prognostic and diagnostic indicator. COMP has also shown promise as a treatment monitor although its natural time course and variations will need to be delineated. The numerous studies on COMP in these different areas will be summarized in this review and the human studies are outlined in Table 1.

## The Disease of Arthritis

As the leading cause of disability in the United States, arthritis is a chronic disease with a significant impact on the population. Based on 2003–2005 data from the National Health Interview Survey (NHIS), an estimated one in five or 46.4 million of U.S. adults have self-reported doctor-diagnosed arthritis. Almost 41% (19 million) of these 46 million adults report limitations in their usual activities due to their arthritis. In addition to activity limitations, 31% (8.2 million) of working age adults with doctor-diagnosed arthritis report being limited in work activities. As the U.S. population ages, these numbers are likely to increase considerably. Each year, arthritis results in 750,000 hospitalizations and 36 million outpatient visits. In 2003, direct medical costs for arthritis were $81 billion while indirect costs were another $47 billion. This economic burden explains the increasing attention that is being directed to arthritis and to finding pharmacological agents to help control the disease.

Arthritis refers to damage to the joints that can be caused by a variety of pathological processes, including osteoarthritis and rheumatoid arthritis. It manifests clinically as abnormal and degraded cartilage, inflamed and thickened synovial tissue, and altered bone structure resulting in pain, decreased mobility, impairment, and disability.^1^–^3^ The diagnosis of arthritis is made based on the patient’s history, physical exam, and radiographs. However, plain radiographs only provide indirect information on cartilage, unlike the direct information it gives on bone. This is because damage to bone can be easily visualized in its actuality on a x-ray while injury to cartilage may only be gleaned from indirect cues such as joint space narrowing, bone erosions or osteophytes, osteopenia or sclerosis, and soft-tissue swelling.^4^,^5^ Serial examinations over several years may be used for assessments of prognosis, treatment, and clinical outcomes. But, sensitivity to change is limited, and clinical manifestations of arthritis do not develop until late in the disease process. By then, the disease process of cartilage degeneration has progressed too far for the chondrocytes to be able to stop or reverse the joint disease, making it too late for early diagnosis and treatment.^6^–^11^ Arthroscopy provides a direct and magnified view of the cartilage surface, but this is an invasive technique that cannot be routinely applied to all patients. Laboratory markers such as erythrocyte sedimentation rate (ESR) and levels of C-reactive protein (CRP) provide useful information about the general inflammation process in some patients, but these markers are not specific to inflammation in joints and correlate poorly with cartilage damage at the individual level.^12^–^15^ Serum IgM rheumatoid factor (RF) and anti-cyclic citrullinated peptide antibodies (anti-CCP) have some diagnostic and prognostic value in the evaluation of rheumatoid arthritis, but positive results can occur with other diseases such as systemic lupus erythematosus, Sjogren’s syndrome, cryoglobulinemia, polymyositis/dermatomyositis, psoriatic arthritis, scleroderma, polymyalgia rheumatica, viral infections, active tuberculosis, tumor, Lyme disease, autoimmune thyroid disease, and palindromic rheumatism.^16^–^28^ By the late stages of arthritis, treatment options are mainly palliative including medications, intra-articular injections, weight loss, ambulatory aids, orthotics, and physical therapy with surgical intervention being the last and most effective option for treatment.

## A Biomarker for Arthritis

There have been recent developments of structure-modifying agents that aim to prevent, delay and stabilize the progress of cartilage damage in osteoarthritis.^29^ Disease modifying anti-rheumatic drugs (DMARDs) that work to suppress the body’s immune system in rheumatoid arthritis to decrease pain and inflammation and to preserve the structure and function of the joints have been successful in treatment^30^ This has lead to an increased interest in identifying a simple and reliable tool to measure cartilage metabolism and the effects of these treatments. It also becomes more important to identify prognostic factors that can predict the evolution of arthritis, especially in the early phases of disease so that these drugs can be started as soon as possible to slow down the progression of the disease. Given the limitations of the tools that are currently available for investigating arthritis, quantitative assessment of biological markers of cartilage degradation and repair would be a promising approach to predict quantitative and dynamic variations in joint remodeling. Since changes in the properties of joint cartilage and loss of matrix components are an integral part of the disease process, biological markers of cartilage metabolism could be used for the early subclinical diagnosis of arthritis. These markers are released into the synovial fluid and eventually to other body fluids, such as blood or urine, where they can be detected. Early diagnosis of impaired cartilage metabolism would enable early treatment, before there is marked loss of articular cartilage and radiographic changes. A biomarker would also be beneficial to evaluate the severity of the disease, to assess and predict the progression of disease, and to monitor effects of treatment. Candidates for potential biologic markers of arthritis include matrix components, cytokines, growth factors, proteases, protease inhibitors, and serum autoantibodies to cartilage components.^31^–^43^ Cartilage oligomeric matrix protein (COMP) is one such potential marker of arthritis that has shown promise as a biomarker of arthritis.

## What is COMP?

Cartilage oligomeric matrix protein (COMP) is also known as thrombospondin 5. It is a 524 kDa homopentameric, extracellular matrix glycoprotein member of the thrombospondin family of calcium-binding proteins. COMP has five identical subunits.^44^ Each subunit consists of 755 amino acids, that interact close to their cysteine-rich aminoterminus, creating a five-stranded coiled coil domain. This is followed by flexible regions of four EGF-like domains and eight TSP calmodulin-like repeats.^45^ The carboxyterminal globular domain binds to collagens I, II, and IX and fibronectin. The function of COMP remains unclear, but it may have a structural role in endochondral ossification and in the assembly and stabilization of the extracellular matrix by its interaction with collagen fibrils and matrix components. COMP has been shown to influence the fibril formation of collagens I and II by promoting early association of collagen molecules thereby accelerating fibrillogenesis with a distinct organization of the fibrils.^46^ It has also been shown that COMP binds to aggrecan, a major component of the cartilage extracellular matrix. This further supports the role of COMP in mediating the matrix molecule interactions needed to organize the cartilage matrix for its load bearing function.^47^ The coiled-coil domain appears to play a role in the storage and delivery of hydrophobic cell-signaling molecules, such as Vitamin D.^48^,^49^ Originally described in cartilage, COMP has also been identified in ligaments, meniscus, tendons, synovium, osteoblasts and vascular smooth muscle.^50^,^51^

COMP is considered a marker of cartilage breakdown, and is being studied as a biological marker in various uses. It has potential as a diagnostic and prognostic indicator and as a marker of the disease severity and the effect of treatment. The first immunoassay for COMP was developed using COMP purified from bovine articular cartilage as an immunogen.^52^ Subsequently, numerous labs have developed both polyclonal and monoclonal antisera to COMP from a variety of species including human.^53^–^57^ With the advent of commercially available enzyme-linked immunosorbent assay (ELISA) kits for COMP, there has been a new resurgence in investigating COMP as a biomarker. While these ELISA kits make it easier to quantitatively measure COMP levels, they do have some differences. These include the species type of COMP to be detected (human or animal), the ELISA method used (sandwich or competitive), the type of antibody used (monoclonal or polyclonal, from different species types), and the labeling of the detection antibody (alkaline phophatase labeled or biotin labeled with streptavidin-HRP conjugate).

## COMP as a Diagnostic Indicator

COMP has been shown to be diagnostic of arthritis and to correlate with the disease severity. In patients with various types of arthritis, COMP levels were detected in all fluids, but were ten times higher in synovial fluid than in serum indicating preferential release from the affected joints. Levels did not correlate with ESR or other acute phase indicators of inflammation.^52^ In the Johnston County Osteoarthritis Project, serum levels of COMP were elevated in participants with osteoarthritis (OA) and increased with the severity of the radiographic knee OA and the numbers of knees and hips that had OA on radiographs.^58^ Serum COMP levels were also higher in patients with bilateral versus unilateral hip involvement in patients with symptomatic hip OA.^59^ Serum COMP levels have also been found to be higher in those with bone scan abnormalities, suggesting that serum concentrations of the markers reflect changes in the turnover in the tissues seen on bone scans.^60^ There have been reports of increased COMP levels in patients with familial OA from a mutation in the type II procollagen gene COL2A1 with a 100% concordance for the development of OA.^61^ Higher serum COMP levels have been observed in patients with radiographic Kellgren-Lawrence Grades III–IV than in Grades I–II lesions,^62^ which may be due to the involvement of more joints in the later stages. Radiographs of the hands, knees, hips, and lateral lumbar spine were obtained, and the OA severity grade for each patient was based on the score for the most severely involved joint.^61^ In a chronic erosive arthritis model in rats, COMP levels correlated with the severity of macroscopically detectable arthritis at two different timepoints: on days 35 and 49 after induction of arthritis with pristane injection.^63^ Serum COMP also highly correlated with degree of histologic cartilage destruction in rats.^64^ In knee OA, serum COMP levels correlated with the clinical manifestation of synovitis, but not with the extent of joint damage.^65^ Serum COMP was found to be a specific marker for the cartilage degradation in RA and not related to the nonspecific inflammatory process, as there was a significant difference in levels when compared to patients with other inflammatory rheumatic diseases with less cartilage-destructive arthritis.^66^ However, sensitivity (15%–48%) and specificity (66%–69%) of COMP as a marker for RA was shown to be low in both selected and unselected cohorts with RA when compared to antibodies against cyclic citrullinated peptides (anti-CCP Ab).^67^

## COMP as a Prognostic Indicator

There has also been evidence supporting COMP as a prognostic indicator. COMP levels were higher in patients with aggressive RA than in those with non-aggressive RA. Patients with aggressive RA were defined as those who required a total hip replacement within four years of disease onset.^68^–^70^ This suggested that COMP might be a prognostic factor for large joint destruction. In other studies, although baseline levels were not predictive of knee joint destruction assessed by changes in joint space width over 5 years, an increase in serum COMP over 1 year^71^ or 3 years^72^ indicated progressive disease in early and established OA. Patients were labelled to have progressive disease if they had a joint space reduction of at least 2 mm on radiograph or if they received surgical intervention. In contrast, the elevation of serum COMP levels at baseline was associated with progression of symptomatic advanced hip OA. This was assessed by changes in radiographic joint space over 1 year measured with digitized image analysis.^59^ In patients with established knee OA, the change in joint space width over 3 years, summed for both knees, correlated positively with serum COMP levels. Patients who progressed by two Kellgren-Lawrence grades^62^ on their radiographs were shown to have had significantly higher COMP levels at baseline as well as at the end of the study.^73^ In another series of patients with knee pain and tibiofemoral OA followed over 5 years, serum COMP was related to progressive joint damage. The serum COMP was higher in those with progression of OA, defined by an increase in joint space narrowing of at least 2 mm or by treatment with total joint replacement. The increased COMP was seen at baseline and each follow-up visit. Logistic regression analysis showed that on average, a 1-unit increase in serum COMP levels increased the probability of radiographic progression by 15%. Serum COMP also rose after joint replacement surgery and remained elevated for up to 12 months.^74^ This suggests that sequential measurements of COMP levels can identify patients at high risk for radiological progression of OA. In patients with traumatic knee injury, it was found that a subgroup with elevated and increasing serum COMP levels were at increased risk for developing posttraumatic osteoarthritis.^75^ In patients with symptoms and clinical signs of hip and knee pathology, but no radiographic evidence OA, a statistically significant association was found between serum COMP and hip-related symptoms, but not knee related symptoms.^76^ This would support the use of serum COMP as a biomarker of hip joint pathology prior to radiographic findings. It is still not clear whether the baseline level or the short-term change in serum COMP levels is a better predictor of joint destruction.

## COMP as a Therapeutic Indicator

In regards to the use of serum COMP to monitor the response to various therapies, there have been varying results. In patients with RA treated with TNF-alpha blockers infliximab or etanercept, serum COMP decreased at 3 months of therapy and remained low at 6 months in responders and non-responders.^77^ Basal levels of COMP in RA patients can predict the extent of clinical response to treatment with adalimumab, another TNF-alpha inhibitor.^78^ Patients with low COMP and CRP levels at baseline were also shown to have a very high American College of Rheumatology (ACR) 70 response rate when treated with various anti-TNF-alpha drugs, in contrast to patients with elevated COMP at baseline.^79^ An ACR 70 response requires a patient to have a 70% reduction in the number of swollen and tender joints, and a reduction of 70% in three of the following five parameters: physician global assessment of disease, patient global assessment of disease, patient assessment of pain, C-reactive protein or erythrocyte sedimentation rate, and degree of disability in Health Assessment Questionnaire score.^80^ The results suggest that COMP level could be helpful in deciding whether continued observation or modification in treatment is warranted in patients who may not be responding to their current treatment early on.^79^ No association between baseline COMP and change in COMP was found with joint space width or scores on the Western Ontario and McMaster Universities Osteoarthritis Index in a study of glucosamine sulfate for OA.^81^,^82^ However, postmenopausal women with rheumatoid arthritis receiving hormone replacement therapy (HRT) had lower serum COMP.^83^ In rats with collagen-induced arthritis treated with corticosteroid therapy, serum COMP levels remained stable compared to increases in COMP over time in placebo-treated rats.^64^ Intra-articular glucocorticoid treatment for knee synovitis in RA patients reduces serum COMP, with a slightly larger decrease of serum COMP in the group randomized to 24 hour bed rest instead of normal activity.^84^ Serum levels of COMP were also used to monitor the therapy response to intravenous bolus steroid therapy in patients with active RA. The intravenous treatment with steroids had a rapid effect on decreasing serum COMP levels within 10 days.^85^ Thus, serum COMP is influenced by different therapies, and may be a valuable parameter for monitoring the treatment response in patients with arthritis.

## Biological Variations of COMP

The normal time course of COMP and its variations will need to be further delineated before it may be used as a widespread biomarker of arthritis. In the Johnston County Osteoarthritis Project, a population-based study of OA in African-Americans and Caucasians, ethnic and gender differences in COMP were not explained by differences in age, BMI, height, presence or severity of radiographic OA, or number of other symptomatic joints. In both ethnic groups, serum COMP increased with age and BMI, and was also higher in those with radiographic OA. African-American women had higher levels of COMP than Caucasian women and Caucasian men had higher COMP than Caucasian women.^86^ In a series of patients in Poland, a correlation was seen between serum COMP levels and age in patients with RA, but not OA. However, in OA patients, there was a correlation between the serum COMP level and Western Ontario and McMaster Universities (WOMAC) index pain scale for the lower limbs and the T-score value of densitometry examinations.^87^ Serum levels of COMP are also affected by exercise, especially during the first 30 minutes. Therefore, samples of blood for analysis of serum COMP should be drawn after at least 30 minutes rest.^88^ Otherwise, during normal everyday activities, serum COMP levels are constant during the day between 8 am and 9 pm. There is a substantial decrease in COMP at night, reaching the lowest levels between 4 and 5 am, suggesting that COMP is eliminated rapidly once it reaches the bloodstream.^89^

## Conclusions

Serum COMP has potential to be used as a biomarker of arthritis. Elevations in this marker have been associated with the presence of arthritis and can correlate with the severity of the disease. Elevated serum COMP levels have also been shown to predict OA progression. Certain therapies aimed at disease modification in OA and RA can influence serum COMP levels, which in turn may reflect cartilage damage. But standards of this marker will need to be established that consider ethnic and gender differences. Further clinical and longitudinal studies of ethnic and other variations in serum COMP and its association with arthritis symptoms and functional status would be of benefit. The metabolism and clearance of COMP, as well as the contributions of non joint tissues to COMP serum levels, will also need to be determined. This information is needed to ascertain if an increase in the level of the marker indicates increased synthesis, increased breakdown or modifications of its clearance. Serum levels reflect the release of COMP from all cartilage or bone structures and elimination at different points in its metabolism. The lack of specificity of COMP for cartilage may limit its use in assessing changes in joint damage in OA/RA. Specific reagents for degraded COMP are lacking, and therefore have limited the usefulness of this marker to determine the presence of arthritis and to develop an assay with a dichotomous outcome (i.e. normal vs. abnormal for a population). In its current form, the assay is useful to monitor response to treatment in a given individual with inflammatory arthritis. The assay may need to be complemented by radiographic or MRI evaluations. However, COMP would be a valuable tool for identifying patients at high risk for rapid joint destruction and for monitoring treatment efficacy.

## Figures and Tables

**Table 1:** Human clinical outcomes in COMP studies.

Study	Year	Disease	No. of patients	Fluid studies	Treatment	Results
Andersson et al.^89^	2006	OA	58	serum		Before exercise or rest, no significant differences in COMP levels were seen. After 60 minutes exercise, serum COMP levels increased (p < 0.001). After 60 minutes of rest the serum levels decreased (p = 0.003). Median serum COMP values in samples obtained prior to exercise or rest at baseline and after 24 weeks did not change between start and end of the study. COMP was increased immediately after exercise (p = 0.018) and had decreased to baseline levels after 30 minutes.
Bleasel et al.^61^	1999	familial OA	47	serum		COMP levels were significantly elevated in Arg 519-Cys mutation-positive individuals (p < 0.001) and increased with OA severity (p < 0.001).
Clark et al.^58^	1999	OA	291	serum		COMP levels of the OA group were significantly higher than those of the control group (p = 0.0093) and also increased significantly with knee OA K/L grade (p = 0.0047), knee OA laterality (p = 0.0043), and number of knee and hip joints involved (p = 0.0001).
Conrozier et al.^59^	1998	OA	48	serum		COMP levels at baseline correlated with hip joint space width at entry and with its yearly mean narrowing (p = 0.002) but not with joint space narrowing grade progression. The concentrations were higher in patients with bilateral hip OA (p = 0.03).
Dragomir et al.^76^	2002	OA	145	serum		Mean Ln COMP was higher among subjects with hip-related clinical signs (p = 0.018), among those with hip-related symptoms (p = 0.046), and among individuals meeting American College of Rheumatology clinical criteria for hip OA (p = 0.021). There were no statistically significant associations between any of the knee-related clinical signs and symptoms and Ln COMP.
Forslind et al.^68^	1992	RA	18	serum		Patients in the aggressive RA group initially had increased levels of COMP, in contrast to normal levels found in the non-aggressive group (p < 0.001).
Jordan et al.^86^	2003	OA	769	serum		Levels of ln COMP were associated with age, BMI, and all definitions of radiographic OA (p = 0.0001), and varied by ethnicity and sex. In adjusted models, ln COMP was higher in African American women than in Caucasian women (p = 0.003) and higher in Caucasian men than Caucasian women (p = 0.0001). There were no statistically significant differences in serum ln COMP levels between African American men and women.
Kuhne et al.^75^	1998	traumatic knee injury	30	serum and synovial fluid		The average COMP level was significantly higher in patients with traumatic knee injury than in the control group at all timepoints. The subgroup with elevated and increasing serum COMP levels, including some with antibodies against cartilage matrix molecules, appeared at increased risk for developing posttraumatic osteoarthritis.The SF and serum levels of COMP correlated with each other.
Mansson et al.^69^	1995	RA	18	serum		Compared with a matched normal population, increased concentrations of cartilage oligomeric matrix protein (COMP) were found in all subjects who developed rapid hip joint destruction.
Nikolaisen et al.^67^	2007	RA, ankylosing spondylitis, noninflammatory conditions	342	serum		The presence of anti-CCP Ab had the highest accuracy in distinguishing RA patients. Sensitivity (15%–48%) and specificity (66%–69%) of COMP as a marker for RA was low.
Petersson et al.^60^	1998a	OA	38	serum		COMP levels were significantly higher in the subjects with bone scan abnormalities (p = 0.02) and correlated positively with the extent of bone scan abnormalities (p = 0.002).
Petersson et al.^72^	1998b	OA	38	serum		Levels of COMP and bone sialoprotein increased significantly (p < 0.001) in the subjects with radiographic OA at follow-up, while remaining unchanged in the subjects with normal radiographs at follow-up.
Saxne and Heinegard^52^	1992	RA, OA, reactive arthritis, juvenile chronic arthritis	155	serum and synovial fluid		COMP SF levels were always higher then in serum paired samples. Highest SF levels were found in reactive arthritis patients and the lowest in RA patients. Serum levels were low in patients with JCA and RA and did not differ otherwise between groups or from controls.
Sharif et al.^71^	1995	OA	94	serum		COMP levels increased during the first year in subjects with progression of knee OA compared to those who did not progress (p < 0.001). COMP increased during the first year in progressive subjects by 5.04 micrograms/ml more than in non-progressive patients.
Sharif et al.^74^	2004	OA	115	serum		The mean COMP level at baseline was significantly higher in the patients with progression of OA compared with the nonprogressors (p < 0.036). COMP levels were higher during periods of radiographic progression and identified periods of progression that were nonlinear. Logistic regression analysis showed that on average, a 1-unit increase in serum COMP levels increased the probability of radiographic progression by 15%.
Skoumal et al.^66^	2004	RA and other inflammatory rheumatic diseases	150	serum		COMP is a specific marker in RA and not related to the nonspecific inflammatory process as elevated COMP levels were detected only in patients with RA and in a few with psoriatic arthritis.
Vilim et al.^65^	2001	OA and synovitis	196	serum		COMP levels were correlated with age, synovitis and an interaction of synovitis and OA severity. Synovitis showed the strongest effect on COMP levels (P < 0.01).
Vilim et al.^73^	2002	OA	48	serum		The change in joint space over 3 years correlated positively with serum COMP level at baseline (p < 0.01) as well as at study end (p < 0.001), when summed for both knees.
Wislowska and Jablonska^87^	2005	RA and OA	60	serum		Correlation between COMP level and the age of RA patients (p < 0.005) and disease activity score (DAS) value (p < 0.01) was found. In OA patients, no correlation was found between the COMP level and patients’ age and disease duration. There was a correlation between the COMP level and Western Ontario and McMaster Universities (WOMAC) index pain scale for the lower limbs (p < 0.005) and T-score value of densitometry examinations (p < 0.036) in OA patients. No statistical differences were found between the average serum COMP level in RA and OA patients.
Wolheim et al.^70^	1997	RA	18	serum		COMP levels at study inclusion were significantly higher in the subjects with early hip joint destruction compared to the patients in the more benign group (p < 0.001).
Crnkic et al.^77^	2003	RA	49	serum	infliximab or entanercept	COMP levels decreased at 3 months in both infliximab- and etanercept-treated patients (P < 0.001 and <0.005, respectively) and remained low at 6 months. There was no significant correlation between changes in or concentrations of serum COMP and serum C-reactive protein at any time point.
Bruyere et al.^82^	2003	OA	212	serum	glucosamine sulfate	The 3-year radiological progression of knee OA could be predicted by a 1-year increase in osteocalcin or a 1 year decrease in hyaluronic acid levels. Eventually, no signficant correlation was observed between 3 year changes in biochemical marker levels and 3 year changes in joint space width (JSW). In the glucosamine sulfate group, no marker at baseline were correlated with the 3 year changes in JSW.
Forsblad d’Elia et al.^83^	2004	RA	88	serum	hormone replacement therapy	Treatment with HRT resulted in decrease in CTX-I (p < 0.001), ICTP (p < 0.001), PICP (p < 0.05), COMP (p < 0.01), and CTX-II (p < 0.05) at 2 years.
Morozzi et al.^78^	2007a	RA	29	serum	adalimumab	Patients with low serum COMP levels (<10 U/l) at baseline showed a significantly (p < 0.02) higher ACR70 response (>50%) than patients with higher COMP levels within 3 months, and also at 6 months.
Skoumal et al.^85^	2006	RA	17	serum	intravenous steroid treatment	In contrast to the reactive arthritis group, COMP levels of RA + patients (p < 0.004) and the visual analog scale (p < 0.0001) decreased significantly within 2–10 days after the first treatment with steroids. The CRP levels remained unchanged in both groups.
Weitoft et al.^84^	2005	RA	20	serum	intra-articular glucocorticoid injection	After the glucocorticoid injection, COMP levels decreased in both 24 hour bed rest and normal activity groups (p < 0.001), but significantly more in resting patients. Serum osteocalcin levels decreased significantly (p < 0.001) without any difference between the groups.

**Abbreviations:** JCA, juvenile chronic arthritis; OA, osteoarthritis; RA, rheumatoid arthritis; SF, synovial fluid; CTX-1, C-terminal telopeptide fragments of type I collagen; ICTP, C-terminal telopeptide of type I collagen; PICP, C-terminal propeptide of type I procollagen; CTX-II, collagen type II C-telopeptide degradation fragments.
